# Development and validation of a MRI-radiomics-based machine learning approach in High Grade Glioma to detect early recurrence

**DOI:** 10.3389/fonc.2024.1449235

**Published:** 2024-11-14

**Authors:** Fabrizio Pignotti, Tamara Ius, Rosellina Russo, Daniele Bagatto, Francesco Beghella Bartoli, Edda Boccia, Luca Boldrini, Silvia Chiesa, Chiara Ciardi, Davide Cusumano, Carolina Giordano, Giuseppe La Rocca, Ciro Mazzarella, Edoardo Mazzucchi, Alessandro Olivi, Miran Skrap, Houng Elena Tran, Giuseppe Varcasia, Simona Gaudino, Giovanni Sabatino

**Affiliations:** ^1^ Department of Neurosurgery, Mater Olbia Hospital, Olbia, Italy; ^2^ Institute of Neurosurgery, Fondazione Policlinico Universitario A. Gemelli IRCCS, Catholic University, Rome, Italy; ^3^ Neurosurgery Unit, Head-Neck and NeuroScience Department, University Hospital of Udine, Udine, Italy; ^4^ Advanced Radiodiagnostics Centre, Unità Operativa Semplice Dipartimentale (UOSD) Neuroradiology, Fondazione Policlinico Universitario Agostino Gemelli IRCSS, Rome, Italy; ^5^ Department of Neuroradiology, Azienda Sanitaria Universitaria Friuli Centrale (ASUFC) “Santa Maria Della Misericordia”, Udine, Italy; ^6^ Department of Radiology, Radiation Oncology and Hematology, Fondazione Policlinico Universitario Agostino Gemelli IRCSS, Rome, Italy; ^7^ Medical Physics Unit, Mater Olbia Hospital, Olbia, Italy; ^8^ Institute of Neurosurgery, Fondazione Policlinico Universitario Agostino Gemelli IRCSS, Rome, Italy

**Keywords:** machine learning, recurrence, high grade glioma, radiomics, prognosis

## Abstract

**Purpose:**

Patients diagnosed with High Grade Gliomas (HGG) generally tend to have a relatively negative prognosis with a high risk of early tumor recurrence (TR) after post-operative radio-chemotherapy. The assessment of the pre-operative risk of early versus delayed TR can be crucial to develop a personalized surgical approach. The purpose of this article is to predict TR using MRI radiomic analysis.

**Methods:**

Data were retrospectively collected from a database. A total of 248 patients were included based on the availability of 6-month TR results: 188 were used to train the model, the others to externally validate it. After manual segmentation of the tumor, Radiomic features were extracted and different machine learning models were implemented considering a combination of T1 and T2 weighted MR sequences. Receiver Operating Characteristic (ROC) curve was calculated with relative model performance metrics (accuracy, sensitivity, specificity, positive predictive value (PPV) and negative predictive value (NPV)) at the best threshold based on the Youden Index.

**Results:**

Models performance were evaluated based on test set results. The best model resulted to be the XGBoost, with an area under ROC curve of 0.72 (95% CI: 0.56 - 0.87). At the best threshold, the model exhibits 0.75 (95% CI: 0.63 - 0.75) as accuracy, 0.62 (95% CI: 0.38 - 0.83) as sensitivity 0.80 (95% CI: 0.66 - 0.89 as specificity, 0.53 (95% CI: 0.31 - 0.73) as PPV, 0.88 (95% CI: 0.72 - 0.94) as NPV.

**Conclusion:**

MRI radiomic analysis represents a powerful tool to predict late HGG recurrence, which can be useful to plan personalized surgical treatments and to offer pertinent patient pre-operative counseling.

## Introduction

Despite recent advances in surgical technology and genetic discoveries, patients diagnosed with High Grade Gliomas (HGG), which is considered as grade 4, generally have a dismal prognosis with high risk of early tumor recurrence (TR) after post-operative radio-chemotherapy (1–4).

Compelling evidence, based on objective tumoral volume analysis, supports the role of the extent of resection (EOR) in HGG patients as the first step of patients management (5, 6).

Recent technological innovations have increased the safety of surgical resection, while expanding surgical options and indications for HGG surgical treatment (7, 8): several techniques can be currently used during the surgical procedure, such as intraoperative ultrasound (iUS), cortical mapping, sodium fluorescein (9) and 5-ALA fluorescence, with the aim of fostering higher rates of total resection and so increasing the survival chance (10–19).

Surgical treatment, however, can be rarely considered as radical, due to the infiltrating tumor nature, its multifocal presentation, and ill-defined tumor margins.

Although the Stupp protocol was introduced as post-operative standard treatment more than 15 years ago, alternative recent approaches have not been developed so far, and the 5-year survival has not significantly changed in these last decades (20).

Unfortunately, the infiltrative growth, the rapid proliferative rate of malignant cells and the appearance of treatment-resistant cell clones shortly after initial therapy tend to recur within 2 cm of resection margins, independently by the initial EOR exhibited by the patient (21). In this challenging setting, assessment of pre-operative risk of early versus delayed TR assumes a crucial role to develop a personalized surgical approach (with respect to surgery versus biopsy).

A presurgical identification of HGG patients with high risk of recurrence after 6 months from surgery may have several advantages (22): first of all, a more aggressive surgical resection may be pursued in patients with low risk of TR, planning the use of all intra-operative tools and strategies that allow a maximal safe resection. Furthermore, deep genetic sequences may be considered to assist clinicians during postsurgical decision-making involving patients with high risk of TR.

In addition, detection of early TR risk should encourage efforts to better understand the role of early intensified bridging therapies for HGG between surgery and postsurgical treatments (23).

Radiomics is a field of medical imaging that focuses on the extraction and advanced analysis of large amounts of quantitative features from medical images called “radiomic features”. The goal of radiomics is to convert medical images into high-dimensional data that can be analyzed to uncover underlying patterns related to disease characteristics, prognosis, and treatment response (24).

The aim of this study is to develop a Machine Learning (ML) model based on radiomics features extracted from MRI images able to stratify the risk of TR (within 6 months) in newly diagnosed HGG and support clinicians in the decision-making process.

## Materials and methods

### Patient population and image data

This retrospective study was focused on patients affected by high grade glioma, which is defined as grade 4, enrolled in two Institutions: Fondazione Policlinico Universitario Agostino Gemelli IRCCS in Rome (FPG) and Santa Maria della Misericordia in Udine (SMM).

The experimental protocol adopted in this study was approved by the Ethical Committee of Policlinico Universitario Agostino Gemelli IRCCS.

The informed consent was obtained for all the patients included in the study.

Patients from FPG were treated from January 2016 to December 2019, patients from SMM from November 2014 to June 2020. Tumor grading was defined considering the 2021 WHO staging (25).

Inclusion criteria were: age >/= 18 years; no previous surgery; no preoperative chemo- or radiotherapy; at least 6 months of follow up; objective evaluation of preoperative tumor volume on MRI images in DICOM format based on post-contrast T1-weighted MRI sequences and T2-weighted MRI sequences; objective estimation of EOR on post-contrast T1-weighted MRI sequences; revision of histopathological specimens by using the new 2021 WHO Classification of Tumors of the Central Nervous System; MGMT promoter methylation and IDH1/IDH2 mutation status assessment.

All the patients were subjected to the following therapeutic approach: one month before the surgical procedure, all the patients were scanned with an MR acquisition, which was carried out using the GE and Philips scanners 1.5 T in Rome and the Siemens scanner 1.5 T in Udine.

Patients with the following criteria were excluded from the study: diagnostic images blurred or some of required sequences missing.

As for surgery, all the patients underwent the following procedure: the surgical technique was carried out with an intraoperative protocol that involved the use of contrast enhancement ultrasounds (CEUS) and the fluorescence of 5-aminolevulinic acid (5-ALA). Neuro-navigation system was used in all cases, while the intra-operative neurophysiological monitoring was performed in all cases of proximity of the tumor to the cortico-spinal tract. Awake surgery was selected for patients that presented HGG in the dominant hemisphere, close to the inferior front-occipital fasciculus (IFOF) or the superior longitudinal fasciculus (SLF). In a subgroup of selected patient (young age, tumor not close to ventricular system) Carmustine Wafers (CWs) were implanted after surgical tumor removal and intra-operative pathological confirmation of HGG. CWs were not utilized when tumor removal required the creation of a large opening of ventricle and/or the basal cistern.

All methods were performed in accordance with the national guidelines and regulations and in accordance with the Declaration of Helsinki.

### Image acquisition

All examinations were performed using a 1.5 T MR scanner (Siemens Aera in Udine and GE—Optima mr450 and Philips-Ingenia in Rome) with an eight-channel head coil. All study protocols included axial T2-weighted TSE/FSE images with a slice thickness between 4 and 5 mm and post-contrast volumetric T1-weighted images MPRAGE/FSPGR/WATS with a slice thickness between 1 and 1.2 mm. Imaging parameters are described in **Table 1**.

**Table 1:** Technical details of MR sequences.

SIEMENS AERA 1,5 T		
Sequence	T1-MPRAGE	T2-TSE
Echo time	2,74 ms	95 ms
NEX	1	2
Repetition time	2200 ms	2380 ms
No. of sections	256	23
Receiver bandwidth	190 Hz/Px	163 Hz/Px
Echo train length	–	28
FOV	250 mm	230mm
Section thickness	1 mm	5 mm
Section spacing	0	1,3 mm
Matrix size	256x232	208x320
Phase direction	RL	RL
PHILIPS Ingenia 1,5T		
Sequence	T1 WATS	T2 TSE
Echo time	6.2ms	100 ms
NEX	1	3
Repetition time	13 ms	3651 ms
No. of sections	155	36
WFS	216.6 Hz/Px	212.3 Hz/Px
TFE factor	195	23
FOV	250mm	240mm
Section thickness	1 mm	4 mm
Section spacing	0	0.4 mm
Matrix size	252x200	513x331
Phase direction	RL	RL
GE Optima 1,5 T		
Sequence	T1 FSPGR	T2 FSE
Echo time	2.2ms	130 ms
NEX	1	2
Repetition time	7.7 ms	3867 ms
No. of sections	150	34
Receiver bandwidth	22.73 Hz/Px	31.25 Hz/Px
Echo train length	–	21
FOV	250mm	240mm
Section thickness	1.2 mm	4 mm
Section spacing	0	0.4 mm
Matrix size	288x288	356x288
Phase direction	RL	RL

### Image pre-processing and radiomic features extraction

**Figure 1:**
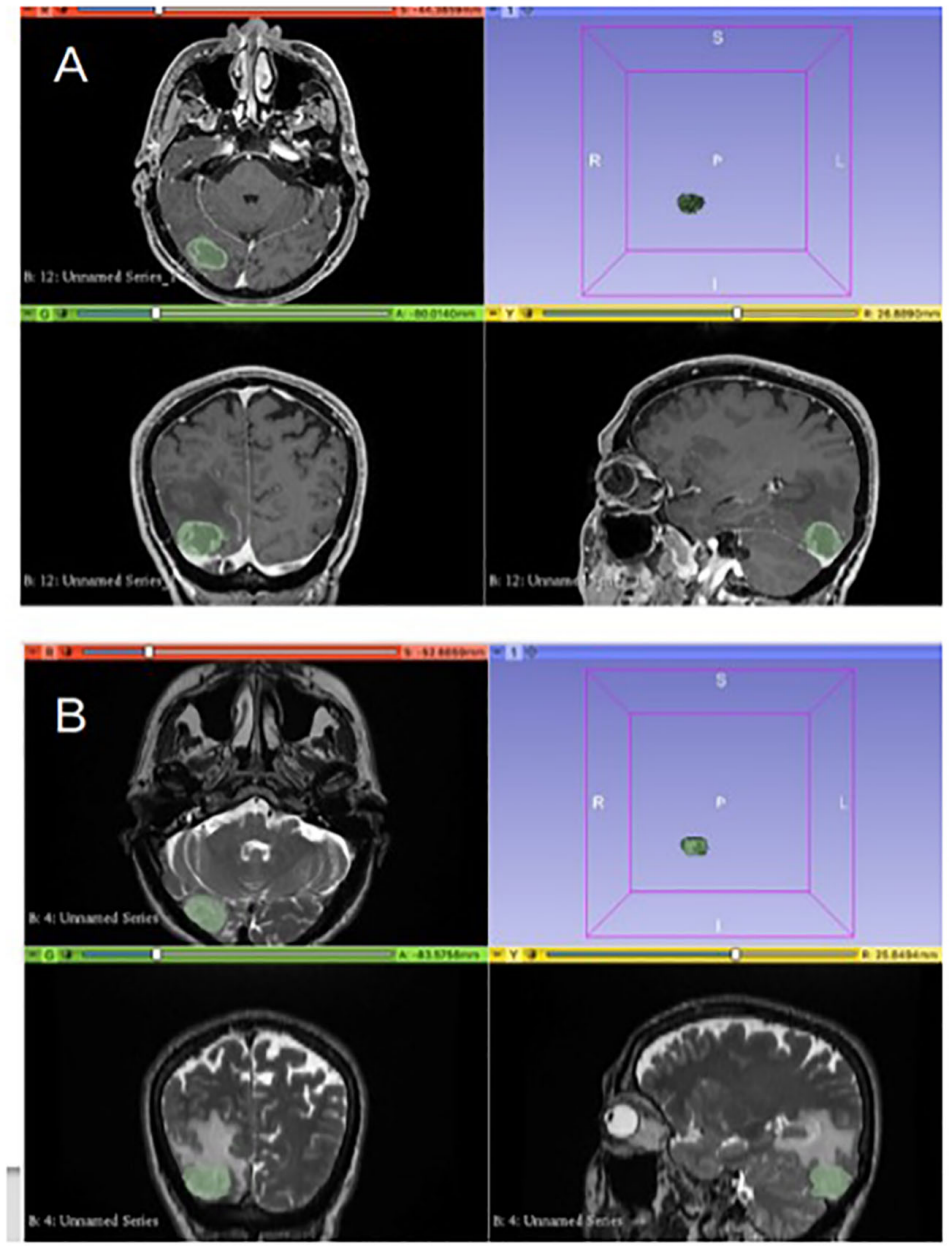
Segmentation of the tumoral areas: in image **(A)**, delineation of the ROI on 3D T1 W post contrast images on the “enhancing” component of the tumor. In image **(B)**, the ROI on axial 2D T2w images is delineated for the solid and infiltrative components of the tumor.

Presurgical MRI performed on 1.5T scanners in FPG and SMM of HGG were analyzed by three neuroradiologists who assessed image quality, excluding patients with images degraded by artifacts or who did not present axial 3D T1 weighted post contrast and axial 2D T2 weighted. Manual segmentation of the tumoral areas was performed by one Neuroradiologist with the software “3D Slicer image computing platform” with ROIs drawn separately on T1 and T2W images as follows: on axial 3D T1 weighted contrast images post-contrast, the ROI on the “enhancing” component of the tumor was delineated, while on axial 2D T2 ROIs were outlined on the solid component of the tumor and the infiltrative one, excluding the frankly edematous areas (with higher signal in T2) (**Figure 1**). Before any analysis, the program anonymizes any DICOM.

Image pre-processing and radiomic analyses were performed using Python 3.7.

Image pre-processing was performed via N4 bias field correction to correct low frequency intensity non uniformity and subject-specific Z-score normalization to normalize MR intensity scales and allow inter-patients comparison.

For each patient, a total of 100 radiomic features (26) were extracted from the specific ROI defined in each pre-processed MR sequence. Features were extracted in their 3D version using PyRadiomics (27).

Extracted features belonged to three families: morphology-based (14), first order (18) and second order (68). For the second order features we used a bin width discretization strategy with a bin width value of 25. Morphology-based features capture geometric characteristics of the ROI. First order features describe statistical properties of the grey level histogram, offering global metrics for the distribution of the grey levels within the ROI. Second order features provide localized measures of grey level distribution within the ROI.

### Radiomic features selection and modeling

The training set included the patients enrolled in Rome, while the test set included the patients enrolled in Udine. We decided to follow this approach to develop a prediction model using data from one hospital and test its generalizability on data from a different hospital, for external testing to pursue a TRIPOD 3 approach (36).

Each radiomic feature was normalized using the z-score in the training set, and the z-score parameters of the training set were used to normalize the features in the test set.

After features extraction, feature selection and radiomics modeling was performed on the training set only. The reproducibility of radiomic features was evaluated with respect to the MRI scanner manufacturer using the t-test. Features that resulted not reproducible (p-value < 0.05) were excluded from the following analysis.

Further feature selection methods were applied to prevent overfitting, reduce linear correlations among variables and obtain a minimal and stable set of radiomic features. These methods included the univariate analysis with the Wilcoxon-Mann-Whitney statistical test (WMW, significance level of 0.05) and the computation of the Pearson correlation coefficient (PCC) with the exclusion of features that were highly correlated with any other feature (PCC > 0.9).

Different machine learning models, namely regularized logistic regression, random forest, XGBoost and Support Vector Machine (SVM) were trained on the selected radiomic features extracted from axial 3D T1w and axial 2D T2w.

The predictive models aimed to address a binary classification problem for the prediction of the 6-months progression free survival: class 0 denoted patients without 6-months TR, class 1 denoted patients with 6-months TR.

For each machine learning model, hyper-parameters tuning was performed with a grid search strategy using 3-fold cross-validation (28).

The developed models were externally evaluated on the testing set in terms of discriminative ability and predictive performance. In particular, the area under the curve (AUC) of the receiver operating characteristics (ROC) curve was calculated, and the model performance metrics based on the classification matrix were computed at the best cut-off threshold, identified by maximizing the Youden Index calculated on the training set. The metrics investigated were accurancy, sensitvity, specificity, positive and negative predictive values (PPV and NPV). 95% confidence intervals (CI) of AUC and classification metrics were computed according to bootstrap (29) and Jeffreys (30) methods, respectively.

## Results

### Patients population

When performing radiomic analysis and modeling, the initial dataset consisting in 273 patients affected by HGG of first diagnosis and undergone respective surgery followed by Stupp protocol, was shrunk to 248 patients on the basis of the availability of the 6-month TR outcome.

Data were split into train (approx. 75%) and test (approx. 25%) sets resulting in 188 patients from Rome and 60 patients from Udine, respectively.

Demographic and clinicopathological characteristics of the included patients are reported in **Table 2**.

**Table 2:** Baseline characteristics of the study population.

Parameters	Trainig set (Rome)	Test set (Udine)	Total
**Initial patients**	206	67	273
**No FU**	18	7	25
**Patients included**	**188**	**60**	**248**
**Mean age** (years)	62.84 ± 4.86	62.52 ± 5.32	62.76 ± 4.98
Sex			
Male	119 (63.3%)	36 (60%)	155 (62.5%)
Female	69 (36.7%)	24 (40%)	93 (37.5%)
Side			
Left	77 (41%)	28 (46.7%)	105 (42.3%)
Right	111 (59%)	32 (53.3%)	143 (57.7%)
Tumor site			
Precentral	63 (33.5%)	18 (30%)	81 (32.7%)
Postcentral	50 (26.6%)	16 (26.7%)	66 (26.6%)
Temporal/insular	75 (39.8%)	26 (43.3%)	101 (40.7%)
5-ALA			
yes	126 (67%)	47 (78.3%)	173 (69.8%)
no	62 (32%)	13 (21.7%)	75 (31.2%)
Biological features			
MGMT methylation (yes vs no)	124 vs 64 (66% vs 34%)	40 vs 20 (66.7% vs 33.3%)	164 vs 84 (66.1% vs 33.9%)
IDH 1/2 mutation (yes vs no)	8 vs 180 (4.3% vs 95.7%)	3 vs 57 (5% vs 95%)	11 vs 237 (4.4% vs 95.6%)
Ki-67 (mean)	24.25 (3-90)	41,2 (5-75)	29 (4-80)
OS			
Alive (yes vs no)	53 vs 135 (28.2% vs 71.8%)	6 vs 54 (10% vs 90%)	59 vs 189 (23.8% vs 76.2%)
Average of FU times (months)	13.4 (0-35)	17.1 (0-29)	14.3 (0-31)
PFS			
**Class 0 (no 6-months TR)**	**109** (58%)	**44** (73.3%)	153 (61.7%)
**Class 1 (6-months TR)**	**79** (42%)	**16** (26.7%)	95 (38.3%)

**Figure 2:**
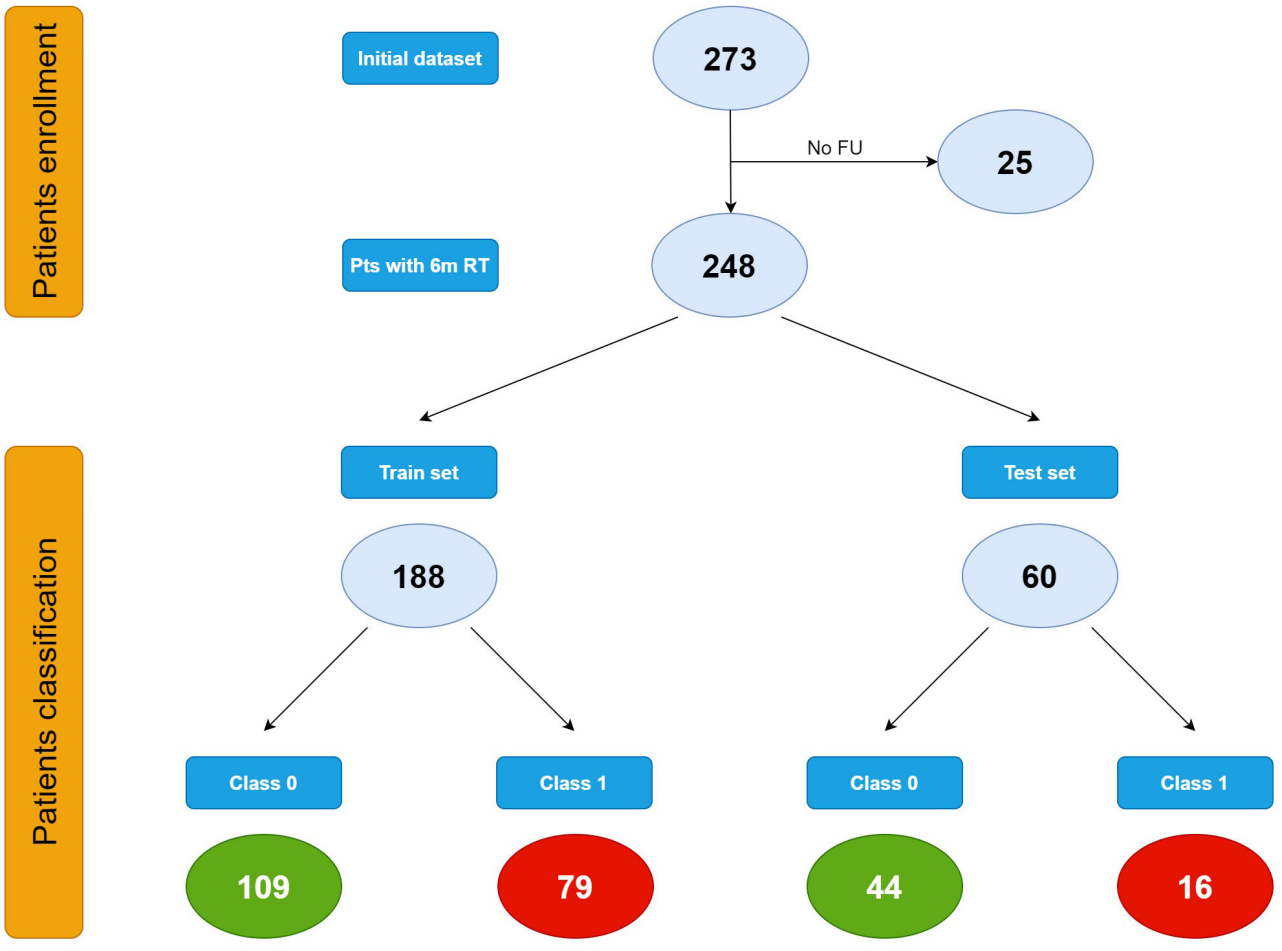
Patients classification.

Characteristics of the study population are described using means ± s.d. (standard deviation) or median and range for continuous variables, number of cases with relative percentages reported in parentheses for categorical variables. Bold values are the corresponding value to **Figure 2**.

(FU, follow-up; 5-ALA, 5-aminolevulinic acid; MGMT, O-6-Methylguanine-DNA Methyltransferase; IDH, Isocitrate dehydrogenase 1; OS, overall survival; PFS, progression free survival; TR, tumor recurrence).

In the training set 109 patients were classified as class 0 and 79 patients as class 1. In the test set, 44 patients belonged to class 0 and 16 to class 1 (**Figure 2**).

### Radiomic features selection

Features selection was applied to features extracted from T1w and T2w sequences.

As for T1w sequence, the t-test evaluating features stability with respect to the manufacturer shrank the number of features from 100 to 58, further reduced by the WMW test and PCC which led to four stable relevant features, reported in **Table 3** with their corresponding WMW test p-values.

**Table 3:** Relevant features resulting from features selection performed on T1w and T2w sequences and corresponding p-values and used for radiomics modeling.

MR sequence	Feature	WMW test p-value
T1w	original_shape_MajorAxisLength	0.02
T1w	original_shape_Maximum2DDiameterColumn	0.03
T1w	original_shape_Maximum2DDiameterSlice	0.04
T1w	original_firstorder_TotalEnergy	0.04
T2w	Original_first_order_kurtosis	0.03

These features belong to morphology-based (“shape”) and first order families.

As for T2w sequence, 85 features out of 100 showed stability with respect to the manufacturer but only one of them was significant at univariate analysis, as reported in **Table 3**.

Thus, **Table 3** included all the features used for the radiomics modeling.

**Figure 3:**
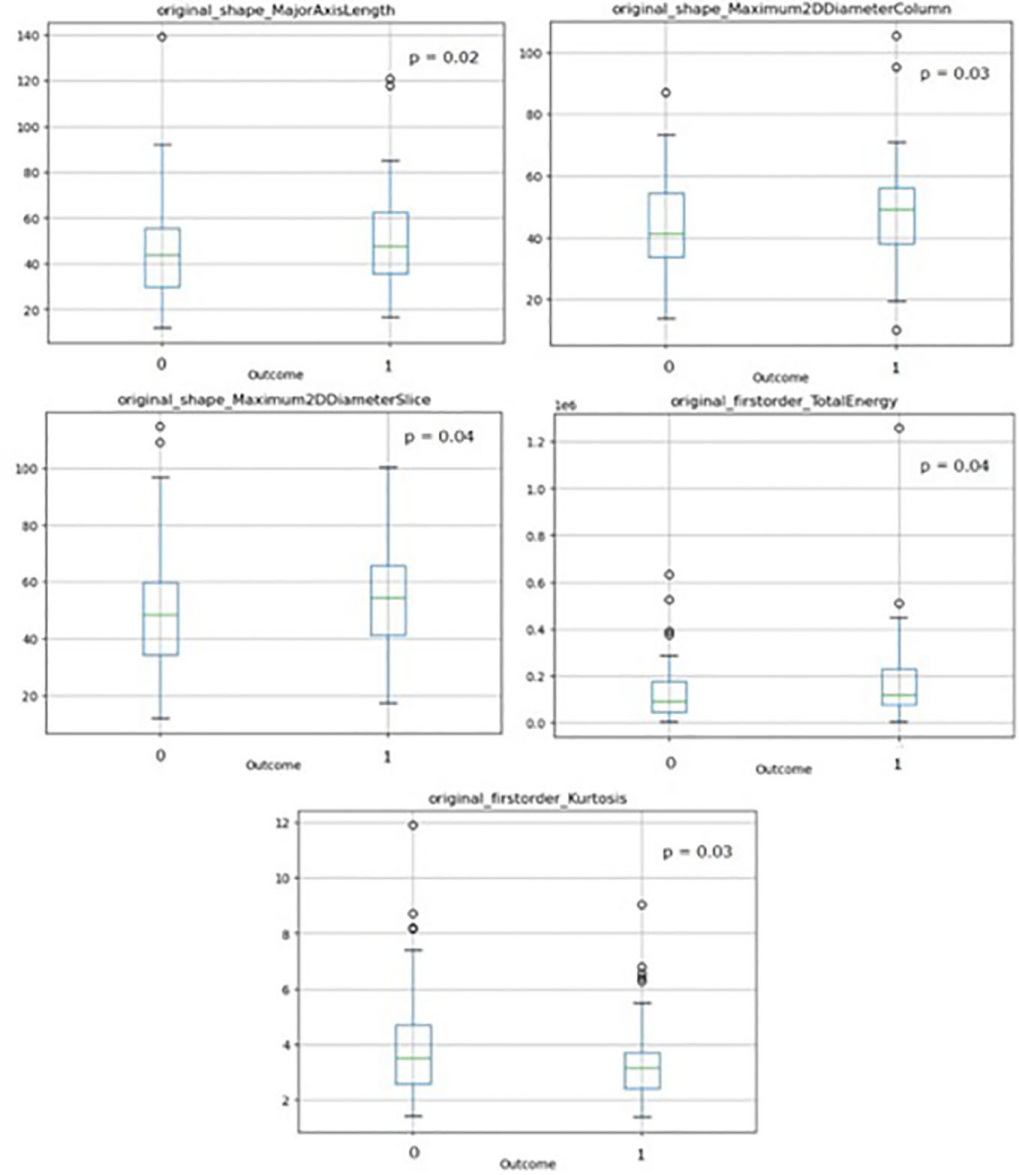
Boxplots of the selected radiomics features used for radiomics modeling showing stability with respect to the outcome with corresponding p-values resulting from the WMW test. (‘1’=patients with 6-months TR, ‘0’=patients without 6-months TR).

Boxplots of the selected features showing stability with respect to the outcome are shown in **Figure 3**, with corresponding p-values resulting from the WMW test.

### Modeling

A combined T1w&T2w (T1wT2w) modeling method was implemented, consisting in grouping the T1wT2w relevant features in a unique input dataset (see **Table 3**).

**Figure 4:**
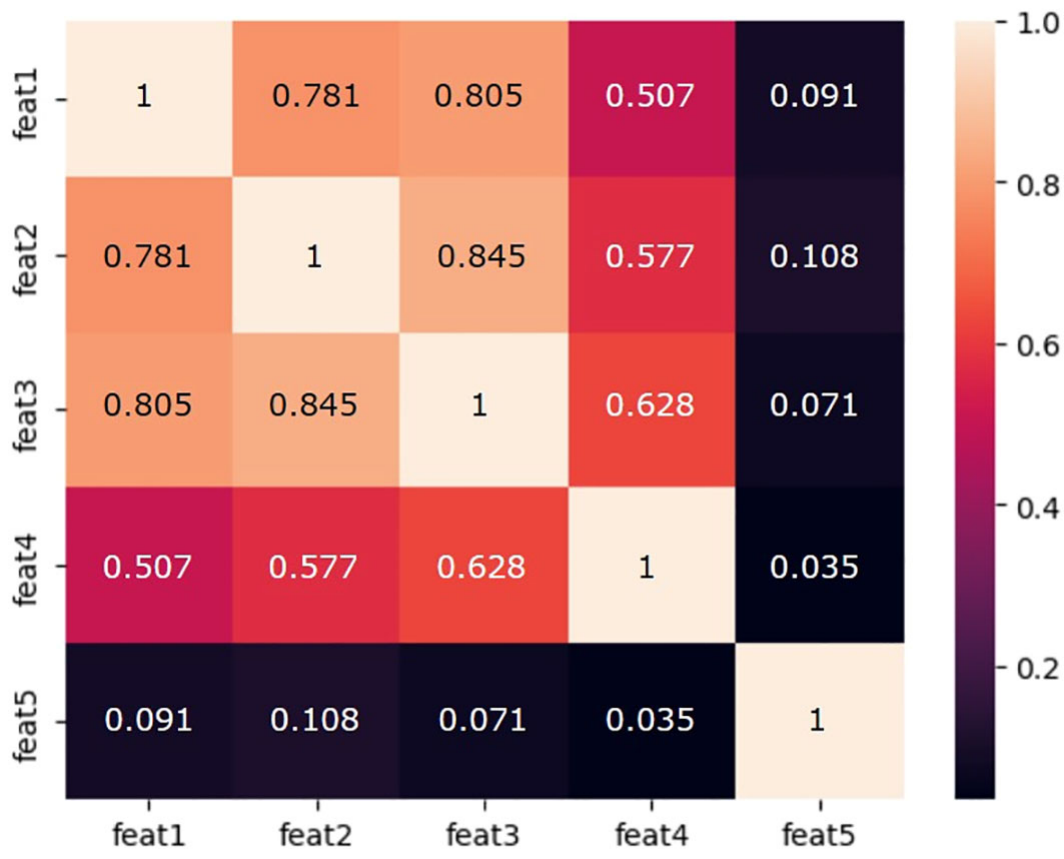
Cross-correlation matrix of the T1wT2w significant features used for radiomics modeling. feat1: original_shape_MajorAxisLength; feat2: original_shape_Maximum2DDiameterColumn; feat3: original_shape_Maximum2DDiameterSlice; feat4: original_firstorder_TotalEnergy; feat5: Original_first_order_kurtosis.

The cross-correlation matrix of the T1wT2w significant features is reported in **Figure 4**.

**Table 4** reports the discriminative and predictive performance metrics for all the implemented ML models trained on T1w and T2w for both training and validation sets.

**Table 4:** Predictive performance metrics for ML models trained on T1w and T2w for training and validation sets.

		AUC	Accuracy	Sensitivity	Specificity	PPV	NPV
**XGBoost**	Training 95% CI	0.68 (0.60- 0.75)	0.66 (0.60 - 0.73)	0.42 (0.31 - 0.53)	0.84 (0.77 - 0.90)	0.66 (0.52- 0.78)	0.67 (0.59 - 0.74)
	Test 95% CI	0.72 (0.56 - 0.87)	0.75 (0.63 - 0.85)	0.62 (0.38 - 0.83)	0.80 (0.66 - 0.89)	0.53 (0.31 - 0.73)	0.85 0.72 - 0.94)
**Regularized Logistic** **Regression**	Training 95% CI	0.63 (0.55 - 0.70)	0.60 (0.54 - 0.67)	0.57 (0.46 - 0.67)	0.63 (0.54 - 0.72)	0.53 (0.42 - 0.63)	0.67 (0.58 - 0.76)
	Test 95% CI	0.52 (0.36 - 0.68)	0.41 (0.30 - 0.54)	0.56 (0.33 - 0.78)	0.36 (0.23 - 0.51)	0.24 (0.13 - 0.40)	0.69 (0.49 - 0.85)
**Random** **Forest**	Training 95% CI	0.99 (0.99 - 1.0)	0.97 (0.95 - 0.99)	0.98 (0.94 - 1.0)	0.97 (0.93 - 0.99)	0.96 (0.90 - 0.99)	0.99 (0.96 - 1.0)
	Test 95% CI	0.51 (0.33 - 0.70)	0.38 (0.27 - 0.51)	0.68 (0.44 - 0.87)	0.27 (0.16 - 0.42)	0.25 (0.14 - 0.40)	0.70 (0.47 - 0.88)
**SVM**	Training 95% CI	0.54 (0.46 - 0.63)	0.60 (0.54 - 0.67)	0.21 (0.14 - 0.32)	0.90 (0.82 - 0.94)	0.59 (0.41 - 0.75)	0.61 (0.53 - 0.68)
	Test 95% CI	0.61 (0.45 - 0.76)	0.68 (0.56 - 0.79)	0.25 (0.09 - 0.49)	0.84 (0.71 - 0.93)	0.36 (0.14 - 0.65)	0.75 (0.62 - 0.86)

Based on the metrics evaluated on the test set, the best model resulted to be the XGBoost, with a test set AUC of 0.72 and 95% CI of 0.56 - 0.87, and an accuracy of 0.75 with a 95% CI of 0.63 - 0.85. This model obtained high values for the specificity equal to 0.80 (95% CI: 0.66 - 0.89), and NPV equal to 0.85 (95% CI: 0.72 – 0.94).

The other models showed the problem of model over-fitting to the training data (i.e. Random Forest) or poor model performance for the training and test data (i.e. regularized logistic regression and SVM).

## Discussion

### Machine learning approach

Today MRI has a primary role in diagnosis, planning and monitoring of HGG patients: clinicians typically use brain MRI to evaluate radiological HGG features such as size, location, edema and enhancement characteristics. MRI features are today not sufficient to predict the risk of recurrence in HGG (31, 32):for this reason, there is a huge need to assess additional imaging biomarkers via computational methods (33, 34).

In the last years, a radiology-based approach focusing on prognosis prediction has gained an important burst fostered by the fast development of advanced computational tools able to manage a significant amount of MRI and clinical data.

Radiomics has recently emerged as a powerful data-driven approach that can offer insights into clinically relevant questions related to diagnosis, prediction, prognosis, as well as treatment response assessment (35).

In this investigation radiomic analysis and modeling were performed with the aim to select the most significant and robust MRI features able to predict which patients affected by HGG at first diagnosis would develop progression within 6 months or later. Prediction performance resulting from training was also externally tested following a TRIPOD 3 study approach, according to the original TRIPOD guidelines (36). Clustering was not accounted for in this study limiting model predictive performance and generalizability (48, 49). Future work may be conducted to evaluate the presence of potential cluster effects.

The 6-months TR time-point was chosen as cut-off value to discriminate patient with early TR from those with later TR for two main clinical reasons: it matches the standard timing of post-operative radiological assessment after conventional postsurgical treatments and it makes homogenous the study populations before the non-standardized rescue therapies at tumor recurrence.

Thus, detecting patients with low risk of TR after 6 months from surgery encourage neurosurgeons to extent tumor resection with the aim of exploiting the most modern intraoperative tools and strategies that allow a maximal safe resection.

We found that, among all features’ families, morphological features show most of the predictive power and that smaller features values decrease the probability to observe 6-month tumor recurrence in patients.

The best model resulted to be the XGBoost (eXtreme Gradient Boosting), an optimized distributed gradient boosting library designed to be highly efficient, flexible and portable. It implements ML algorithms under the Gradient Boosting framework, providing distributed gradient-boosted decision tree (GBDT). Boosting is an ensemble learning method that combines a set of weak base estimators into a strong learner to minimize training errors. For these reasons, XGBoost is considered to be one of the leading ML libraries for regression, classification and ranking problems.

Modeling results are reported in **Table 3**: the developed XGBoost showed a high value of specificity, which reflects the model ability in identifying patients with lower risk to experience tumor recurrence within 6 months and that might undergo a more aggressive surgical resection.

High values of specificity were observed in training (84%) and test set (80%). Features selection applied to this study highlighted that morphological features hold most of the predictive power in discriminating patients with positive and negative outcome. Although it is hard to find a direct biological interpretation of these findings, it is worth mentioning that radiomic features are not relevant if considered only “per se”. It is actually important to relate them to the model context and take linear and non-linear interactions between variables into account. In this light, a possible explanation can be given assuming that changes in morphological features might reflect tissues structural alterations (e.g. shape, volume etc…) and be more related to tumor developments. On the other hand, it is also important to ensure that selected features describing the model are actually stable, non-redundant and independent from noise or other non-relevant variables.

Pre-operative estimation of HGG biological behavior could help clinicians in detecting cases that could benefit from a maximal safe resection (e.g. HGG patients with pre-operative estimated of low risk of early TR).

On the other hand, pre-operative estimation of high risk of early TR (especially in elderly patients) could drive the choice to biopsy rather than surgery (37).

In addition, for patients with higher risk of TR, multiple tissue samplings should be extracted in order to investigate *ad hoc* target therapies related to lesions’ high spatial heterogeneity (38).

Our results are aligned with the study performed by Li et al. (39) with AUC of 0.70 in the training set for the prediction of disease progression at 6 months that used radiomics features extracted from multiple MRI sequences (T2 and FLAIR).

Other studies using smaller sample sizes developed MRI-based radiomics models for the prediction of the progression-free survival in patients with glioblastoma obtaining similar results. Choi et al. (40) obtained an integrated time-dependent AUC of 0.62, while Bathla et al. (41) achieved a C-index up to 0.64.

This investigation presents several limitations. First of all, this was a retrospective study including only two centers: further studies with more heterogeneous datasets would be an interesting point for future development.

Radiomic features reproducibility can be strongly affected by image acquisition parameters and scan protocols, which can vary widely across and within institutions. We took this heterogeneity into account and in order to evaluate features robustness among different centers, we performed a t-test analysis with respect to MRI scanners manufacturers. This allowed to exclude unstable features. In this light, to minimize data variability, harmonization methods might be introduced, as proposed by several research groups which focused on different modified ComBat algorithms (42–46). Although being very powerful, radiomic analysis offers no insights toward biological interpretation of the achieved findings and this study also shows this limitation: many efforts have been made in these recent years to reintroduce biological meaning into radiomics. However, some recent studies also suggest that biological correlation with radiomic features is not mandatory (47).

In addition, considering the integration of features and clinical variables in a clinical-radiomic model could help improving models’ performance in predicting 6-months TR.

Lastly, we did not assess the overall survival rate for this cohort. Data regarding selection criteria adopted at TR to plan the salvage treatment were not available. Each patient underwent an individualized management at TR, so we have not developed standardized protocols for treatments at TR. With our sample size, we did not have the statistical power to tease out the survival rate for patients undergoing different treatments. A future multi-center prospective study of HGG recurrence will be necessary to properly assess survival rate according to the salvage treatment adopted at TR.

The high incidence of early TR should encourage efforts to better understand the role of early intensified bridging therapies for HGG between surgery and postsurgical treatments.

In conclusion, the methodology adopted in this investigation is extremely time-consuming and makes it unsuitable for clinical daily implementation. The next step in this field, beyond increasing accuracy and simplifying the workflow, will be the development of an open source, easily scalable and efficient artificial intelligence algorithm requiring simple or null external intervention from physicians.

## Conclusions

A thorough and reliable ML-model based on combined T1w&T2w sequences to detect the lower risk of TR in newly diagnosed HGG was trained and validated on external cohort.

Our results confirm the potential role of pre-operative MRI analysis beyond the classical anatomical and morphological parameters. MRI radiomic analysis represents a powerful tool to predict early HGG recurrence, to plan personalized surgical treatment and to offer patients pre-operative counseling. In the future, a prospective multicenter study with a larger sample size is needed in order to validate our results, to optimize prediction models for clinical practice, and to overcome the intrinsic limitations of retrospective studies met so far.

## Data Availability

The original contributions presented in the study are included in the article/supplementary material. Further inquiries can be directed to the corresponding author.
